# Effectiveness of Colchicine or Canakinumab in Japanese Patients with Familial Mediterranean Fever: A Single-Center Study

**DOI:** 10.3390/jcm12196272

**Published:** 2023-09-28

**Authors:** Shuhei Yoshida, Yuya Sumichika, Kenji Saito, Haruki Matsumoto, Jumpei Temmoku, Yuya Fujita, Naoki Matsuoka, Tomoyuki Asano, Shuzo Sato, Kiyoshi Migita

**Affiliations:** Department of Rheumatology, Fukushima Medical University School of Medicine, 1 Hikarigaoka, Fukushima 960-1295, Japannaoki-11@fmu.ac.jp (N.M.);

**Keywords:** Familial Mediterranean Fever, interleukin-1, canakinumab, colchicine-resistant, *MEFV* mutation

## Abstract

**Background:** To investigate the clinical features of Japanese patients with Familial Mediterranean Fever (FMF), we evaluated the frequency of attacks, treatment responses, and adverse effects in 27 patients with FMF treated with colchicine or canakinumab in a real-world clinical setting. **Methods:** We retrospectively reviewed 27 Japanese patients with FMF treated at our institute between April 2012 and June 2023. All patients were diagnosed with FMF according to the Tel-Hashomer criteria. We performed genetic analyses of the *MEFV* gene using targeted next-generation sequencing. The clinical response was monitored through the number of attacks, and inflammatory markers were monitored through the C-reactive protein (CRP), and serum amyloid A (SAA) concentrations. Colchicine resistance was defined as the presence of at least one attack/month despite administration of the maximum tolerated dose of colchicine for at least 6 months, and C-reactive protein and serum amyloid A levels above the normal range between attacks. **Results:** A total of 27 patients diagnosed with FMF were enrolled in this study and the median follow-up period was 36.4 months. The median attack frequency was 1.0 (interquartile range: 0.33–1.0) every 3 months before treatment initiation. All the patients (n = 27) were treated with colchicine. Among the 27 patients, 20 (71.8%) showed a clinical response and 7 (25.9%) showed an incomplete response with sufficient doses of colchicine (n = 5) and non-sufficient doses (n = 2). Two patients on non-sufficient doses were unable to increase colchicine to the maximum dose due to diarrhea and liver dysfunction. All seven patients achieved a reduction in attack frequency after the initiation of canakinumab. No serious adverse events associated with canakinumab treatment were observed. In these seven patients with colchicine-resistant FMF (crFMF), the *MEFV* exon 10 variant was not detected, and the absence ratio of the *MEFV* variant was significantly higher compared to those without crFMF. **Conclusions:** Colchicine was effective in 71.8% (20/27) of Japanese patients with FMF; however, the remaining patients (7/27) had crFMF. Canakinumab effectively controlled febrile attacks in crFMF, even in the absence of pathogenic *MEFV* exon 10 variants.

## 1. Introduction

Familial Mediterranean Fever (FMF) is a hereditary autoinflammatory disorder characterized by acute attacks of fever and serosal inflammation [1]. Colchicine, the mainstay of FMF treatment, has been widely considered safe and effective in reducing the frequency of febrile attacks in most patients with FMF [2]. However, approximately 10% of Japanese patients with FMF continue to experience frequent inflammatory episodes despite receiving sufficient doses of colchicine or because they cannot take colchicine at all [3]. This condition is known as colchicine resistance and colchicine intolerance. Patients with chronic inflammation despite colchicine treatment may be at risk of developing AA amyloidosis, which is the most serious complication of FMF [4].

In Mediterranean populations, the *MEFV* exon 10 mutation p.Met694Val has been demonstrated to be associated with FMF severity and increases the risk of AA amyloidosis [5,6]. Although a genotype–phenotype correlation has been reported in Japanese patients with FMF [3], the relationship between *MEFV* genotypes and FMF severity, including colchicine resistance, has not been elucidated [3]. Additionally, the phenotypic heterogeneity of Japanese patients with FMF may suggest the presence of other modifier genes or environmental factors in addition to *MEFV* mutations [7].

Interleukin (IL)-1 inhibitors are treatment options for patients with colchicine-refractory or intolerant FMF [8,9]. These inhibitors include canakinumab, which is an anti-IL-1β monoclonal antibody [10], anakinra, which is an IL-1 receptor inhibitor [11], and rilonacept, which is an IL-1 trap [12]. Canakinumab was approved in Japan in December 2016 and has been used for patients with colchicine-resistant or colchicine-intolerant FMF. However, information on their effectiveness in the prevention of febrile attacks and safety data under therapeutic guidelines are still lacking for Japanese patients. Thus, this study aimed to evaluate the genetic and clinical manifestations and effectiveness of therapeutic interventions, including colchicine and IL-1 inhibitors, in Japanese patients with FMF.

## 2. Materials and Methods

### 2.1. Patients

Patients diagnosed with FMF according to the Tel-Hashomer diagnostic criteria [13] at the Department of Rheumatology of Fukushima Medical University Hospital between April 2012 and June 2023 were enrolled. The doctor in charge recorded epidemiologic data (sex, consanguinity of parents, familial history, and age at onset of signs of inflammation) and main clinical data (fever; thoracic, abdominal, articular, and cutaneous signs; duration and frequency of episodes; and response to colchicine) using a standard form. A diagnosis of FMF was made if the patient had the major criteria and two or more minor criteria of the Tel-Hashomer criteria [13]. On the basis of the Tel-Hashomer criteria, we divided the study subjects into two groups: typical and atypical FMF [13]. Patients with Typical FMF had typical episodes of peritonitis, pleuritis, monoarthritis, or fever alone, as specified by the Tel-Hashomer criteria. Atypical patients with FMF had an “incomplete” attack, which was considered incomplete if it differed from the definition of a typical attack in only 1 or 2 of the following features: temperature less than 38 °C; attack duration longer or shorter than specified periods (12 h to 3 days), but not shorter than 6 h or longer than a week; no signs of peritonitis during an abdominal attack, or signs were localized; atypical distribution of arthritis [3,13]. This study was approved by the ethical committees of Fukushima Medical University (No. 2020-110).

### 2.2. Clinical Course and Treatment Outcomes

The FMF attacks were confirmed by the presence of fever, clinical findings of serositis/arthritis, and elevated CRP levels. The details of each attack (duration, type, severity, and maximum body temperature) were recorded.

Patients were considered to have colchicine-resistant FMF (crFMF) if they recorded one or more attacks with CRP and SAA levels above the normal range between attacks each month despite receiving the maximally tolerated dose of colchicine for at least 6 months [14]. Colchicine resistance was defined as the presence of at least one attack/month despite administration of the maximum tolerated dose of colchicine for at least 6 months, and C-reactive protein and serum amyloid A levels above the normal range between attacks. Colchicine intolerance was defined as the inability to take colchicine at all due to adverse events. All patients with crFMF received the first dose of canakinumab. The treatment period started with the first injection, and the patients received subcutaneous injections of canakinumab (150 mg) at 4-week intervals. The canakinumab dose could be increased to 300 mg if an attack occurred between the first doses; however, none of the patients received an increased dose of canakinumab. All adverse events and laboratory values were recorded at each visit to assess the safety of canakinumab treatment.

### 2.3. MEFV Gene Analysis

*MEFV* gene analysis was performed on all patients in this study. The QIAamp DNA blood kit (QIAGEN, Venlo, The Netherlands) was used to extract genomic DNA from the blood samples. Genetic analysis of the entire exon of *MEFV* was done via targeted next-generation sequencing using the MiSeq platform [15].

### 2.4. Statistical Analysis

Data are presented as medians and interquartile ranges for continuous variables and as frequencies and percentages for qualitative variables. Fisher’s exact probability test and the Mann–Whitney U test were used to compare differences between groups. Wilcoxon signed-rank tests with Bonferroni correction were used to evaluate the pre- and post-treatment changes. Statistical significance for all tests was defined as a two-tailed *p*-value < 0.05. Statistical analyses were performed using the SPSS Statistics software (version 25.0; IBM Corp., Armonk, NY, USA).

## 3. Results

### 3.1. Patients’ Baseline Characteristics

In total, twenty-seven patients with FMF who met the Tel-Hashomer criteria were enrolled. All patients were diagnosed with complete- (typical FMF) or incomplete-type FMF (atypical FMF). Baseline patient demographics and clinical characteristics are shown in Table 1. Of the 27 patients included in this study, 16 (59.3%) were female and 11 (40.7%) were male. The median age at disease onset was 30 years (interquartile range (IQR): 19.5–40.5 years), with a typical FMF preponderance (17/27; 63.0%). The median duration from FMF onset to diagnosis was 4.0 years (IQR: 1.5–9.0 years). The median serum attack-free C-reactive protein (CRP) level was 0.09 (IQR range: 0.06–0.55) mg/dL, and the median serum amyloid A (SAA) level was 4.10 (QR range: 3.48–4.88) μg/mL, both within the reference ranges. Genetic analysis results were available for all the patients with FMF. No homozygous variants of the *MEFV* gene were identified. Heterozygous variants were detected in 11 cases: E148Q (n = 5), M694I (n = 1), R354Q (n = 1), S503C (n = 1), R503C (n = 1), G304R (n = 1) and R202Q (n = 1). Compound–heterozygous variants were detected in 13 cases: E148/L110 (n = 3), P369S/R408Q (n = 3), M694I/E148Q (n = 2), M694I/E148Q/L110P (n = 1), E148Q/G304R (n = 1), E148Q/P369S (n = 1), E148Q/R408Q/P158S (n = 1) and E148Q/L110P/P369S/R408Q (n = 1). Three cases had no *MEFV* gene variants. The five autoimmune comorbidities included vascular Behçet’s disease (n = 1), spondyloarthritis (n = 1), hyperthyroidism (n = 1), mixed connective tissue disease (n = 1), and ulcerative colitis (n = 1).

### 3.2. Comparison of Colchicine Responsiveness between crFMF and Non-crFMF Patients

**Figure 1:**
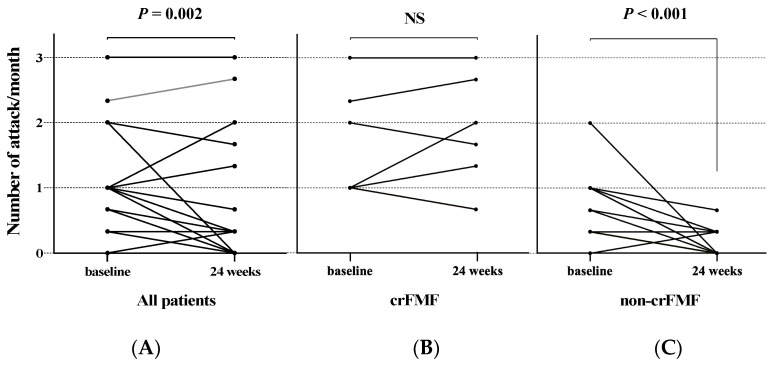
Attack frequency before and after colchicine administration in patients with FMF attack frequency for all patients with FMF (**A**), the crFMF group (**B**), and the non-crFMF group (**C**). The mean number of fever attacks during the 6 months before and after colchicine administration for all patients with FMF is shown. cr: colchicine-resistance; FMF: Familial Mediterranean Fever.

All patients were initiated on colchicine treatment after being diagnosed with FMF. All patients with FMF were able to continue receiving colchicine. The mean dose of colchicine was 0.98 mg/day (SD: 0.44). Colchicine resistance was observed in seven of these patients (25.9%). Five of the seven patients had persistent febrile attacks despite the maximum dose of colchicine, and two were unable to increase colchicine to the maximum dose due to diarrhea and liver dysfunction. The enrolled patients with FMF were divided into two groups: those with colchicine-resistant FMF (crFMF) and those with non-colchicine-resistant FMF (non-crFMF). The *MEFV* gene variants, frequency of febrile attacks before and after colchicine administration, and serum SAA and CRP levels during the interictal period were compared between the crFMF and non-crFMF groups. Table 2 compares the clinical characteristics of both groups. Attack frequency after colchicine administration was significantly higher in the crFMF group (*p* < 0.001). Regarding *MEFV* gene variants, there were significantly more exon 2 variants in the non-crFMF group (*p* = 0.011), and significantly more cases with no variants in the crFMF group (*p* = 0.012). A comparison of colchicine responsiveness in terms of attack frequency before and after colchicine administration in each group is shown in Figure 1. A significant reduction in attack frequency was observed in the non-crFMF group after colchicine administration (*p* < 0.001). The response to colchicine was poor in the crFMF-treated group.

### 3.3. Clinical Characteristics of Patients with crFMF and Clinical Response to Canakinumab

**Figure 2:**
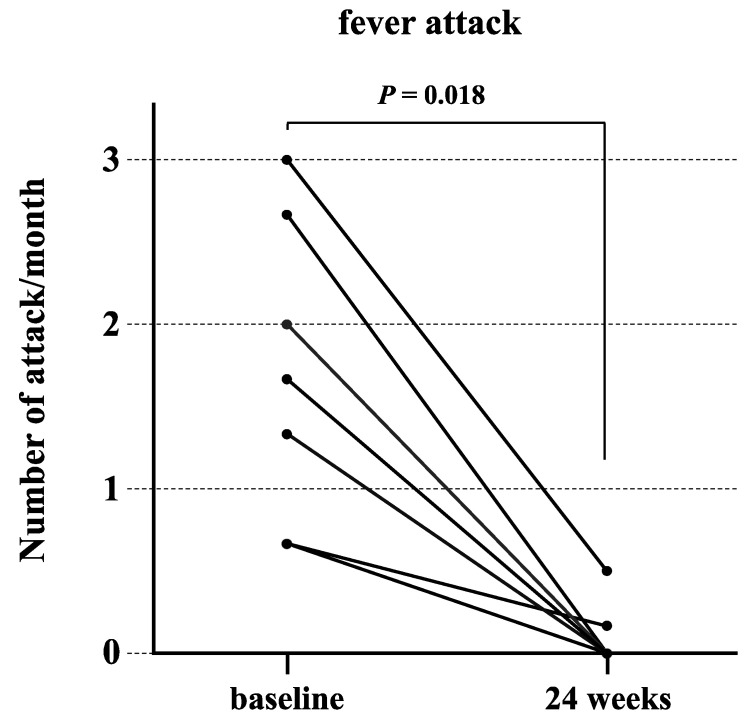
Attack frequency before and after the introduction of canakinumab for patients with crFMF. The mean number of fever attacks during the 6 months before and after canakinumab administration for colchicine-resistant FMF patients is shown. The mean number of seizures per month 24 weeks after canakinumab treatment was significantly reduced compared to baseline (*p* = 0.018).

**Figure 3:**
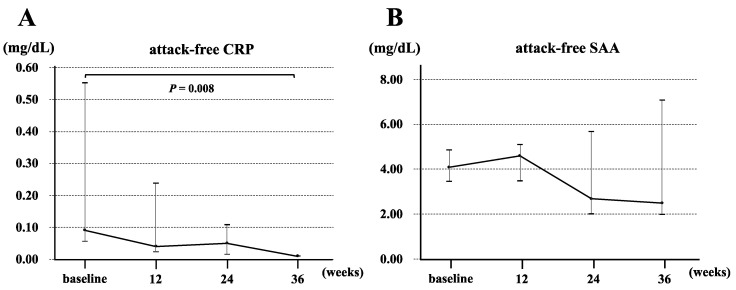
Changes in serum CRP (**A**) and SAA (**B**) levels during the interictal period. Dots show the median, and bars indicate IQR. CRP: C-reactive protein; SAA: serum amyloid A.

Canakinumab was administered to patients with crFMF according to the European League Against Rheumatism (EULAR) recommendations for the management of FMF [14]. There were no adverse events with canakinumab and all patients were able to continue treatment. Figure 2 shows the frequency of fever before and after administration of canakinumab in the crFMF groups. The frequency of febrile attacks significantly decreased after canakinumab administration (*p* = 0.018). Figure 3A,B show the median values of serum attack-free CRP and serum amyloid A (SAA) over time, respectively. Serum CRP levels during the interictal period decreased significantly (*p* = 0.008) before canakinumab treatment compared to 36 weeks after the start of treatment. No statistically significant differences in serum SAA levels during the interictal period were detected before or after treatment compared to any time point after treatment.

## 4. Discussion

Interleukin (IL)-1 antagonists are treatment options for patients with colchicine-resistant or -intolerant FMF [8,9]. Canakinumab, a therapeutic anti-interleukin-1β monoclonal antibody, is effective in controlling and preventing febrile attacks in patients with colchicine-resistant FMF (crFMF) [10]. In this study, we present the genetic and clinical characteristics of 27 Japanese patients with FMF who were diagnosed and treated at a single center. We evaluated the clinical efficacy of colchicine and canakinumab in Japanese patients with FMF, including those with crFMF. Interestingly, favorable clinical responses were demonstrated after colchicine treatment initiation in all patients with FMF carrying the M694I variant, which was exclusively observed as an exon 10 variant in Japanese patients [3,16]. In contrast, in FMF patients carrying non-exon10 variants or no *MEFV* variant, colchicine effectiveness was insufficient, and these colchicine-resistant FMF patients (n = 7) were treated with canakinumab. A favorable clinical response was observed after the initiation of canakinumab treatment in these seven patients treated with canakinumab. However, in one female patient with FMF, canakinumab was switched to tocilizumab (TCZ) because she presented with chronic arthritis despite the complete resolution of the febrile attack. In this case, arthritis and febrile attacks were well controlled with TCZ. The IL-6 blockade may be relevant because IL-1 induces IL-6 transcription and increases IL-6 levels [17].

A favorable clinical response was demonstrated in patients with colchicine-resistant FMF even in the absence of *MEFV* non-exon 10 variants, after the initiation of canakinumab [9]. Regarding safety, no adverse events of canakinumab were observed. Our data suggest that IL-1β inhibition using canakinumab is a useful therapeutic option for patients with FMF with an inadequate response to colchicine. Additionally, a significant decrease in serum acute-phase proteins (CRP and SAA) was observed during the non-attack period after the introduction of canakinumab.

A correlation between the *MEFV* genotype and the FMF disease phenotype has been reported in Japanese patients with FMF [3]. However, the severity of FMF, including colchicine resistance, may vary among affected individuals with the same *MEFV* variant, suggesting the presence of several modifiers, including other genes and epigenetic and environmental factors [18]. Although the association of the *MEFV* exon 10 variant M694V with the severity of FMF, including colchicine resistance, has been demonstrated [19], Japanese patients with FMF treated with canakinumab due to colchicine resistance did not carry the *MEFV* exon 10 variant M694I. Additionally, the ratio of patients without MEFV variants was significantly higher in patients with colchicine-resistant FMF (crFMF) than in those without crFMF.

In our study, the exon 10 *MEFV* variant M694I was not associated with colchicine resistance. In contrast, Japanese FMF patients without *MEFV* variants (n = 3) were associated with colchicine resistance, and these patients were treated with canakinumab. In accordance with our results, Tomokawa et al. reported that Japanese patients treated with canakinumab due to colchicine resistance or intolerance did not possess an *MEFV* exon 10 variant [20]. Therefore, environmental factors may affect disease severity or colchicine resistance in Japanese patients with FMF. *MEFV*-encoded pyrin is an important component of multiprotein inflammasome platforms, which regulate caspase 1 activity and the processing of Pro-IL-1β into active forms [21,22]. Decreased RhoA GTPase activity caused by bacterial toxin such as TcdB results in a RhoA-mediated pyrin-specific inflammasome platform and proteolytic activation of IL-1β [23]. However, the role of FMF-associated exon 10 variants in RhoA-induced pyrin inflammasome activity remains unclear. Our data suggest that nongenetic factors, including environmental factors, contribute to colchicine resistance in Japanese patients with FMF without pathogenic *MEFV* variants.

The goals of FMF treatment are to improve quality of life, reduce febrile attacks, and prevent organ damage [14]. Reducing the frequency of FMF attacks in patients who do not respond to colchicine is essential to avoid complications of persistent inflammation, including renal failure due to secondary amyloidosis [14,24]. The results of this study suggest that IL-1β inhibition with canakinumab is a useful therapeutic option for patients with FMF showing an inadequate response to colchicine.

The limitations of this study were the relatively small number of patients and short follow-up periods. However, it is difficult to study FMF in a large number of patients, since FMF is a rare disease. Second, a possibility existed that symptoms unrelated to FMF were mistakenly determined as FMF attacks or that FMF attacks were evaluated as symptoms of other diseases.

## 5. Conclusions

Colchicine resistance was observed in FMF patients carrying non-exon10 variants or no *MEFV* variants but not in those carrying the exon 10 *MEFV* variant. Patients with colchicine-resistant FMF have been successfully treated with canakinumab. Our data suggest that canakinumab is an effective treatment option for Japanese patients with crFMF without *MEFV* non-exon 10 variants. Further investigation is warranted to reproduce these unique therapeutic courses in Japanese patients with FMF.

## Figures and Tables

**Table 1:** Demographic and clinical characteristics of patients with FMF at baseline.

Variables	n = 27
Typical FMF, n (%)	17 (63.0)
Male, n (%)	11 (40.7)
Age at FMF onset (years), median (IQR)	30 (19.5–40.5)
Age at colchicine introduction (years), median (IQR)	39 (29–47)
Duration from FMF onset to diagnosis (years), median (IQR)	4.0 (1.5–9.0)
Use of colchicine, n (%)	27 (100)
Dose of colchine (mg/day), average (SD)	0.98 (0.44)
Colchicine resistance, n (%)	7 (25.9)
Attack frequency (number of attacks per month), median (IQR)	1 (0.33–1)
Attack-free CRP (mg/dL), median (IQR)	0.11 (0.04–0.22)
Attack-free SAA (μg/dL), median (IQR)	3.6 (2.5–6.8)
*MEFV* variants, n (%)	
E148Q/normal	5 (18.5)
E148Q/L110P	3 (11.1)
P369S/R408Q	3 (11.1)
M694I/E148Q	2 (7.4)
M694I/normal	1 (3.7)
M694I/E148Q/L110P	1 (3.7)
E148Q/G304R	1 (3.7)
E148Q/P369S	1 (3.7)
E148Q/R408Q/P158S	1 (3.7)
E148Q/L110P/P369S/R408Q	1 (3.7)
R354Q/normal	1 (3.7)
S503C/normal	1 (3.7)
R503C/normal	1 (3.7)
G304R/normal	1 (3.7)
R202Q/normal	1 (3.7)
No variant	3 (11.1)
Follow-up period (months), median (IQR)	36.4 (11.7–71.1)
Autoimmune comorbidities, n (%)	5 (18.5)

All data are expressed as mean values (SD), median (IQR), or numbers (percentages). IQR: interquartile range; SD: standard deviation; FMF: Familial Mediterranean Fever; CRP: C-reactive protein; SAA: serum amyloid A.

**Table 2:** Comparisons of clinical features between crFMF group and non-crFMF group.

Characteristics	All Patients		
	crFMF (n = 7)	Non-crFMF (n = 20)	*p* Value
Typical FMF, n (%)	5 (71.4)	12 (60.0)	0.678
Male, n (%)	4 (57.1)	7 (35.0)	0.633
Age at colchicine introduction, † years	43 (42–46)	36.5 (28–47.3)	0.431
Duration from FMF onset to diagnosis, † years	5.0 (3.5–11)	3.5 (1.0–9.0)	0.431
Dose of colchine (mg/day), average (SD)	1.14 (0.38)	0.93 (0.46)	0.174
Attack frequency before initiation of colchicine, † number of attacks per month	1.0 (1.0–2.17)	0.66 (0.33–1.0)	0.004 *
Attack frequency 6 months after initiation of colchicine, † number of attacks per month	1.67 (1.0–2.33)	0.0 (0.0–0.1)	<0.001 *
Attack-free CRP, † mg/dL	0.09 (0.06–0.55)	0.11 (0.03–0.21)	0.882
Attack-free SAA, † μg/day	4.1 (3.48–4.88)	3.3 (2.5–6.85)	0.779
*MEFV* Exson 10 variants, n (%)	0 (0)	4 (20.0)	0.545
*MEFV* Exson 2 variants, n (%)	2 (28.6)	17 (85.0)	0.011 *
*MEFV* Exson 3 variants, n (%)	2 (28.6)	4 (20.0)	0.633
*MEFV* Exson 5 variants, n (%)	0 (0)	2 (10.0)	1.00
No variant	3 (42.9)	0 (0)	0.012 *
Follow-up period, † months	51.8 (31.8–86.5)	34.6 (10.7–64.3)	0.370
Autoimmune comorbidities, n (%)	2 (28.6)	3 (15.0)	0.580

† Values are the median with interquartile range. All data are expressed as mean values (SD), median (IQR), or numbers (percentages). IQR: interquartile range; SD: standard deviation; cr: colchicine-resistance; FMF: Familial Mediterranean Fever; CRP: C-reactive protein; SAA: serum amyloid A. * means there is a significant difference at *p* < 0.05.

## Data Availability

The raw data supporting the conclusions of this article will be made available by the authors, without undue reservation.

## Abbreviations

FMF	Familial Mediterranean Fever
crFMF	colchicine-resistant Familial Mediterranean Fever
AA	amyloid A
IL	interleukin
CRP	C-reactive protein
IQR	interquartile range
SAA	serum amyloid A
EULAR	European League Against Rheumatism
TCZ	tocilizumab
